# Volume and diagnosis: an approach to cross-border care in eight European countries

**DOI:** 10.1136/qshc.2008.029553

**Published:** 2009-01-26

**Authors:** P Vallejo, R Suñol, B Van Beek, M J M H Lombarts, C Bruneau, F Vlček

**Affiliations:** 1 Avedis Donabedian Institute, Autonomous University of Barcelona, and CIBER Epidemiology and Public Health (CIBERESP), Barcelona, Spain; 2 Avedis Donabedian Institute, Autonomous University of Barcelona, Barcelona, Spain; 3 European Society for Quality in Healthcare, Limerick, Ireland; 4 Academic Medical Center, Department of Social Medicine, University of Amsterdam, Amsterdam, the Netherlands; 5 Haute Autorité de Santé, Paris, France; 6 Spojená Akreditační Komise, Czech Republic

## Abstract

**Objectives:**

Mobility of patients is a pertinent issue on the European Union’s agenda. This study aimed to estimate the volume and main diagnoses of cross-border care in eight European countries, in order to provide policy makers with background information about the nature of patient mobility in Europe.

**Methods:**

This article reports the combined findings from three independent studies that compiled self-reported information on admissions data and main diagnoses from more than 200 hospitals in eight European countries.

**Results:**

The average volume of cross-border patients accounted for less than 1% of total admissions in the hospitals studied here. Diseases of the circulatory system (mainly acute myocardial infarction) and fractures were the most common reasons for hospitalisation of European patients abroad. Deliveries and other diagnoses related to pregnancy, pneumonia, appendicitis and other diseases of the digestive system, aftercare procedures, and disorders of the eye and adnexa were also common diagnoses for this population.

**Conclusions:**

Hospitals should reinforce their efforts to adapt the care provided to the needs of foreign patients in treatment areas that cover the most frequent pathologies identified in this population.

This study forms part of the Methods of Assessing Response to Quality Improvement Strategies (MARQuIS) project. The objectives of the MARQuIS project are to research and compare different quality improvement policies and strategies in healthcare systems across the member states of the European Union (EU), and to consider their potential use when patients cross borders to receive healthcare. This research was intended to enable an evaluation of the need for formal quality procedures at the EU level for healthcare services, and to support the development of such procedures.^1^

Several studies have been published recently regarding cross-border care, some of them covering different aspects of patient mobility within the EU,^2^ ^3^ regulations to access services in other member states,^4^ common cross-border cooperation involving hospitals from a European perspective,^5^ ^6^ and specific agreements between two or more countries for some treatments.^7^^–^9 Although these studies have provided information to help understand the cross-border phenomenon, very few of them included data on the volume and type of care provided, and when such data has been provided it is mostly fragmentary. The few studies that have provided general data about volume considered that patient mobility is increasing, and stated that the current volume of patient mobility seemed to be relatively low (accounting for around 1% of overall public expenditure on healthcare).^10^ Despite these previous studies, we are still missing basic descriptive information about the characteristics of the cross-border care in Europe. The present study aimed to estimate the volume and type of cross-border care in some European countries, and the findings provide policy makers with background information about the nature of patient mobility.

## MATERIALS AND METHODS

This article combines the findings from three independent studies, each with its own objectives, population, sampling criteria and data-gathering tool. Two of them, the purposive sample study and the Catalan case study, were conducted as exploratory studies in the beginning of 2005. The third one, the MARQuIS questionnaire study, was conducted during the summer of 2006 to gather more detailed information, and to verify the preliminary findings from the two previous studies.

We have operationally considered volume of care as the number of patients admitted to a hospital for a given period of time, and the number of patients treated in the emergency department for a given period. We studied type of care according to the patients’ main diagnosis, coded by the *International Classification of Diseases* (ICD) versions 9 or 10. When the ICD10 was used, the diagnoses were re-coded to ICD9 by the research team. For the analysis, single diagnoses were grouped using the *ICD-9-CM* list of three digit categories.^11^

### Purposive sample study

The aim of this study was to estimate the most common diagnoses in European cross-border care. We analysed volume of admissions and diagnosis data for the year 2003 from hospitals in six of the countries involved in the MARQuIS field test: Belgium, Czech Republic, France, the Netherlands, Poland and Spain. Data were retrieved from the hospitals’ health information systems. The purposive sample procedure included 26 hospitals, selected by MARQuIS country coordinators (researchers from each country), who were asked to identify four hospitals known or assumed (based on available national data) to treat a large number of patients from other European countries. As a data-gathering tool, we developed and piloted the “Review of National Statistics on Health Services to Patients from other European Countries” questionnaire. The questionnaire collected information on hospitals’ activity and patients’ most frequent diagnoses, both for the total population and for foreign EU patients. It also collected activity data about the total population and European foreigners treated in the emergency department. Data were gathered during February and March, 2005.

### Catalan case study

In another exploratory study we recorded the number of admissions and diagnosis data for the year 2003 from the region of Catalonia (Spain). Data were retrieved from the Minimum Data Set (Conjunto Mínimo Básico de Datos, CMBD) of the healthcare information system of the Catalan Public Hospitals network. The analysis was based on all foreign admissions to all Catalan hospitals during 1 year. As a data-gathering tool we used the questionnaire developed for the purposive sample study, and data were obtained in February 2005.

For the purposive sample and the Catalan study we analysed the differences in type of care for cross-border versus the general population. The most common diagnoses from all hospitals were collated for two groups for each study: European foreigners and the remaining general population. The differences in the incidence of each of the most frequent diagnoses for these two subpopulations were analysed with a hypothesis test for two proportions from independent groups.

### MARQuIS questionnaire study

The MARQuIS questionnaire study aimed to measure quality improvement strategies that hospitals in Europe apply, and how well these strategies satisfied cross-border care quality criteria. A total of 1531 hospitals from eight European countries were invited to participate in this study, the inclusion criteria being acute care hospitals with more than 100 beds, and delivering care for at least two of the three selected diagnoses. Hospitals were selected by random sampling, and were stratified into two groups: hospitals known to deliver cross-border care (according to country coordinators), and those that potentially delivered this type of care. Data were collected in 2006 through the web-based MARQuIS questionnaire, a 199-item data-gathering tool that also requested information on the 10 most common diagnoses for foreign European patients admitted to the hospital during 2004. The questionnaire also explored whether the hospitals had agreements in place to deliver care to cross-border patients.^12^

Each hospital provided the list of the 10 most common diagnoses for foreign EU patients in rank order. To analyse this information, the research team weighted the rank of each hospital, so that the diagnosis ranked in position 1 (most frequent) in any hospital was multiplied by 10, the diagnosis ranked in position 2 was multiplied by 9, a rank of 3 was multiplied by 8, and so on until the rank position of 10, which was not multiplied by any factor. The products were summed for each diagnosis to produce total weighted ranks for the group of hospitals. The diagnoses were then ranked in descending order from the highest total weighted rank to the lowest.

## RESULTS

In the purposive sample study we received 17 completed questionnaires from the hospitals, for a response rate of 70%. Six countries were represented: Belgium (2 hospitals), Czech Republic (4), France (2), the Netherlands (3), Poland (4) and Spain (2). Some of the hospitals that completed the questionnaire were not able to provide all the data requested, so the analysis included data from 17 hospitals for admissions, and from 11 for the emergency unit.

The Catalan case study included all data from the Catalonian regional public network, which includes 69 hospitals funded by the regional government.

In the MARQuIS field test we received completed questionnaires from 389 hospitals in eight countries: Belgium (25 hospitals), the Czech Rep (44), France (78), Ireland (25), the Netherlands (10), Poland (80), Spain (113), and the United Kingdom (14), which represented an overall response rate of 25.4%. However, not all the hospitals could provide all the data requested, so volume data for the present analysis were derived from 182 hospitals for admissions and 138 for emergency departments, and diagnosis data for the present analysis were derived from 146 hospitals.

### Type of European cross-border care

Few hospitals (7.7%) participated jointly with another EU hospital to deliver cross-border patient care. However, nearly 24.8% of the hospitals were considering such collaboration. Poland (48.7%) and the Czech Republic (41%) seemed most keen to initiate cross-border collaboration, whereas in France only 7.5% of the hospitals were considering such collaboration. Further, 24.2% (87) of all hospitals had formal financial arrangements with healthcare financiers, and of these hospitals, 41.4% reported that quality requirements were part of the arrangement. Table 1 summarises this information.

**Table 1:** Cross-border care collaboration between hospitals and financiers

	Cross-border care collaboration between EU hospitals			Formal financial arrangements with financiers% (absolute number)
	Formal collaboration exists% (absolute number)	Collaboration is being considered% (absolute number)	No collaboration% (absolute number)	
France	4.5 (3)	7.5 (5)	71.6 (48)	Yes = 3.0 (3)
				No = 85.1 (57)
				DN/NA = 11.9 (8)*
Spain	3.6 (4)	18.9 (21)	72.1 (80)	Yes = 20.0 (22)
				No = 71.8 (79)
				DN/NA = 8.2 (9)
Poland	7.7 (6)	48.7 (38)	38.5 (30)	Yes = 26.0 (20)
				No = 46.8 (36)
				DN/NA = 27.3 (21)
Czech Rep	7.7 (3)	41 (16)	51.3 (20)	Yes = 71.8 (28)
				No = 23.1 (9)
				DN/NA = 5.1 (2)
UK	8.3 (1)	16.7 (2)	66.7 (8)	Yes = 16.7
				No = 58.3
				DN/NA = 25
Ireland	20.8 (5)	8.3 (2)	62.5 (15)	Yes = 25.0 (6)
				No = 66.7 (16)
				DN/NA = 8.3 (2)
Belgium	17.4 (4)	21.7 (5)	56.5 (13)	Yes = 26.1 (6)
				No = 65.2 (15)
				DN/NA = 8.7 (2)
The Netherlands	22.2 (2)	11.1 (1)	66.7 (6)	Yes = 12.5 (1)
				No = 62.5 (5)
				DN/NA = 25 (2)
Total	7.7 (28)	24.8 (90)	60.5 (220)	Yes = 24.2 (87)
				No = 62.2 (224)
				DN/NA = 13.6 (49) (missing n = 123)

*DN/NA, don’t know/no answer.

All the hospitals included in the purposive sample study provided the 10 most common diagnoses for foreign European patients admitted to the centre during 2003. The 10 most common diagnoses from all hospitals together accounted for 3756 patients, whereas the total number of foreign EU patients admitted to these hospitals during the year was 9835. Consequently, 38% of the foreign European patients admitted to these hospitals were represented in the list of main diagnoses. The most common reason for hospitalisation of European foreigners was ischaemic heart disease, followed by two diagnostic groups related to deliveries: normal delivery and complications occurring mainly in the course of labour. Of the 10 most common diagnostic groups, three were related to pregnancy and delivery, three to different kinds of fractures and two to the circulatory system. Appendicitis and “persons seeking health services for specific procedures and aftercare” were also among the most common diagnostic categories for this group of hospitals.

The Catalan Public Hospitals Network admitted 1503 foreign European patients during 2003. The Catalan Public Health Information System provided data for the 15 most frequent diagnoses, grouped into eight diagnostic groups for this population, which accounted for 493 patients. Consequently, 33% of all European foreign patients were included in this list of diagnoses. The main reason for admission of European foreigners was fracture of the lower limb, followed by ischaemic heart disease and appendicitis. Of the eight most common diagnostic groups, three were related to the circulatory system, and three to different types of fractures. Pneumonia and appendicitis were also near the top of the list of most frequent diagnoses. Table 2 shows the results of these two preliminary studies.

**Table 2:** Preliminary studies of types of cross-border care

	Purposive sample study			Catalan case study	
	Main diagnosis		Number of patients	Main diagnosis	Number of patients
1	Ischaemic heart disease (410–414)		789	Fracture of the lower limb (820–829)	102
2	Normal delivery, and other indications for care in pregnancy, labour, and delivery (650–659)		433	Ischemic heart disease (410–414)	84
3	Complications occurring mainly in the course of labour and delivery (660–669)		296	Appendicitis (540–543)	59
4	Fracture of lower limb (820–829)		239	Fracture of the upper limb (810–819)	34
5	Other forms of heart disease (420–429)		215	Pneumonia and influenza (480–487)	32
6	Complications mainly related to pregnancy (640–648)		165	Cerebrovascular disease (430–438)	29
7	Persons seeking health services for specific procedures and aftercare (V50–V59)		153	Other forms of heart disease (420–429)	27
8	Fracture of the upper limb (810–819)		101		
9	Appendicitis (540–543)		100		
10	Fracture of the skull (800–804)		87		
	Number of patients included		3756	Number of patients	1503

Based on these results, the project team selected acute myocardial infarction, appendicitis and deliveries as the three diagnoses to be included in the next step of the MARQuIS project: a hospital questionnaire survey and external assessment of quality improvement. These three diagnoses were chosen as the most common ones in the preliminary studies, and also as the diagnoses that could provide a wide range of information among different hospital services (eg, the medical unit, surgery, emergencies and the maternity unit). Fracture of the lower limb was one of the most common diagnoses, however, the research group decided to use appendicitis; although both require emergency care, appendicitis is a specific condition presenting low case variability and was therefore easier to study. The target group of patients with fractures was more heterogeneous owing to a variety of types of fracture (tibia and fibula, hip, etc.), and would have been more complex to analyse.

Regarding the MARQuIS questionnaire, we calculated the global rank for the group of 146 hospitals that provided data on the most frequent primary diagnoses for foreign European admissions. The 17 weighted rank positions are shown in table 3, which lists the 15 most common diagnoses plus two others, included because of their relevant positions in the preliminary rankings from the Catalan and purposive sample studies. According to the MARQuIS questionnaire ranking, ischaemic heart disease was the most frequent diagnosis among hospitalised European foreigners. In fact, including ischaemic heart disease, four diseases of the circulatory system were among the 10 most frequent diagnoses (the others were: other forms of heart disease, hypertensive disease, and cerebrovascular disease). There were also four diagnoses from the injury and poisoning group in these rank positions, with three different types of fracture and superficial injuries. Two diagnoses for three other groups of pathologies were also included in the ranking: pregnancy, childbirth and puerperium (normal delivery was selected for the MARQuIS project); diseases of the digestive system (including appendicitis, also selected for this study); and diseases of the respiratory system.

**Table 3:** Ranking of the most common diagnoses for European cross-border hospitalisations

Most common diagnostic groups	Rank position in MARQuIS questionnaire	Rank position in purposive sample	Rank position in Catalan case study
Ischaemic heart disease (410–414)	1	1	2
Other forms of heart disease (420–429)	2	5	7
Fracture of the skull (800–804)	3	10	
Fracture of the lower limb (820–829)	4	4	1
Other diseases of the digestive system (570–579)	5		
Hypertensive disease (401–405)	6		
Persons seeking health services for specific procedures and aftercare (V50–V59)	7	7	
Superficial injury (910–919)	8		
Disorders of the eye and adnexa (360–379)	9		
Cerebrovascular disease (430–438)	10		6
Normal delivery, and other indications for care in pregnancy, labour, and delivery (650–659)	11	2	
Chronic obstructive pulmonary disease and allied conditions (490–496)	12		
Diseases of other endocrine glands (250–259)	13		
Appendicitis (540–543)	14	9	3
Pneumonia and influenza (480–487)	15	8	5
Complications mainly related to pregnancy (640–648)	16	6	
Fracture of the upper limb (810–819)	17		4
Complications occurring mainly in the course of labour and delivery (660–669)		3	

### Volume of European cross-border care

We defined the volume of EU foreign care as the proportion of European cross-border patients of all the patients treated in any given institution. For inpatient admissions, data were obtained from all three studies. In the purposive sample of 2003, the percentage of foreign European patients out of the entire population admitted to hospitals accounted for 2.4% of the total population hospitalised. In the Catalan case study, foreign European patients represented 0.21% of all admissions in 2003 (714 404 inpatient admissions). No data were available on the distribution of this population among the 69 hospitals included in the study. Of the 389 hospitals that completed the MARQuIS field test questionnaire, only 182 provided data on the volume of foreign EU patient admissions. This group of hospitals indicated that 0.64% of the total population admitted during 2004 was from another European country.

For the emergency department, data were available from a more limited number of hospitals in both the purposive sample and the MARQuIS questionnaire study. No data were available from the Catalan healthcare system on foreign patients treated in emergency departments. The percentage of foreign Europeans treated for emergency care during 2003 in the hospitals included in the purposive sample accounted for 8.55% of the total population treated in these hospitals. Of the 389 hospitals that completed the questionnaire from the main study, only 138 provided data on the volume of foreign EU patients treated in the emergency unit; 1.04% of the population treated in the emergency unit in these hospitals was from another EU country. Table 4 presents the most relevant data regarding the volume of European cross-border care for both inpatient admissions and emergencies.

**Table 4:** Estimated volume of European cross-border care for inpatient admissions and emergency care

	Inpatient admissions			Emergencies	
	Purposive sample study	Catalan case study	MARQuIS questionnaire study	Purposive sample study	MARQuIS questionnaire study
Number of hospitals that provided data	17	69	182	11	138
Number of EU foreigners hospitalised	12 584	1503	30 731	18 184	56 012
Total number of patients hospitalised	526 540	714 404	481 156	212 686	5 396 435
Percentage of EU patients out of the total number of admissions to the hospital	2.39%	0.21%	0.64%	8.55%	1.04%
Number of hospitals with no EU foreign admissions	0	No data	19	0	19
Number of hospitals with >0 to <1% of EU foreign admissions	10	No data	139	2	96
Number of hospitals with >1 to <5% of EU foreign admissions	3	No data	17	6	20
Number of hospitals with >5% of EU foreign admissions	4	No data	7	3	3
Range of percentage of EU patients out of the total number of admissions	0.01–22.3%	No data	0–88.24%	0.1%–9%	0–11.38%

The MARQuIS questionnaire also explored the volume of foreign Europeans hospitalised for the three pathologies selected for this study because they appeared to be among the most prevalent for this population. Although the overall percentage of foreign Europeans among total admissions in participating hospitals was 0.64%, this percentage was higher for the three pathologies selected for individual analysis: 1.97% for acute myocardial infarction, 1.70% for appendicitis and 1.62% for deliveries.

### Cross-border versus general care

Table 5 summarises the results of the analysis of differences in the incidence of each of the most frequent diagnoses in the local versus foreign populations in the purposive sample and the Catalan study. In the latter, European foreign patients and the general population shared only two of the 10 most common diagnoses (deliveries and ischaemic heart disease). There differences between groups was statistically significant (p<0.05) for almost all diagnoses analysed except malignant neoplasm of the female breast. In the purposive case study, four diagnoses (fracture of the lower limb, deliveries, other forms of heart disease and general symptoms) were on the list of most frequent diagnoses for both groups. The differences between groups was statistically significant (p<0.05) for all diagnoses analysed except for these four. This comparison needs to be viewed with caution since data for only the top 10 diagnoses from each hospital were available, and a valid comparison would have required data from the complete database.

**Table 5:** Preliminary studies of the main diagnoses for foreign admissions compared with the total population

Purposive sample study					Catalan case study			
Most frequent diagnoses		EU foreign admissions incidence (CI)	Total hospitalisations excluding EU incidence (CI)	p	Most frequent diagnoses	EU foreign admissions incidence (CI)	Total hospitalisations excluding EU incidence (CI)	p
Fracture of the lower limb (820–829)		9.05% (7.83% to 10.81%)	2.00% (1.96% to 2.02%)	<0.05	Normal delivery and other indications for care in pregnancy, labour and delivery (650–659)	9.15% (8.58% to 9.73%)	4.64% (4.58% to 4.69%)	<0.05
Ischaemic heart disease (410–414)		5.59% (4.48% to 6.87%)	1.22% (1.19% to 1.24%)	<0.05	Ischaemic heart disease (410–414)	8.02% (7.49% to 8.57%)	3.03% (2.98% to 3.07%)	<0.05
Appendicitis (540–543)		3.93% (3.00% to 5.03%)	0.75% (0.73% to 0.77%)	<0.05	Fracture of the lower limb (820–829)	2.42% (2.12% to 2.74%)	0.03% (0.02% to 0.03%)	<0.05
Pneumonia and influenza (480–487)		2.13% (1.46% to 2.99%)	1.37% (1.34% to 1.39%)	<0.05	Other forms of heart disease (420–429)	1.25% (1.04% to 1.49%)	0.22% (0.21% to 0.23%)	<0.05
Normal delivery, and other indications for care in pregnancy, labour and delivery (650–659)		2.00% (1.35% to 2.83%)	1.81% (1.77% to 1.83%)	0.289	Radiotherapy and chemotherapy	1.21% (1.00–1.44%)	–	–
Cerebrovascular disease (430–438)		1.93% (1.29% to 2.75%)	0.96% (0.93% to 0.98%)	<0.05	Appendicitis (540–543)	1.02% (0.82% to 1.23%)	0.03% (0.03% to 0.03%)	<0.05
Other forms of heart disease (420–429)		1.80% (1.18% to 2.60%)	1.93% (1.90% to 1.96%)	0.351	Cerebrovascular disease (430–438)	0.78% (0.61% to 0.97%)	–	–
Disorders of the eye and adnexa (360–379)		0.13% (0.01% to 0.48%)	6.86% (6.79% to 6.91%)	<0.05	Fracture of the upper limb (810–819)	0.70% (0.54% to 0.88%)	–	–
Chronic obstructive pulmonary disease and allied conditions (490–496)		1.26% (0.76% to 1.96%)	2.24% (2.20% to 2.27%)	<0.05	Malignant neoplasm of bone, connective tissue, skin and breast (170–176)	0.47% (0.34% to 0.62%)	0.58% (0.55% to 0.59%)	0.0763
Hernia of the abdominal cavity (550–553)		0.7% (0.00% to 0.37%)	1.83% (1.80% to 1.86%)	<0.05	Other diseases of respiratory system (510–519)	0.45% (0.32% to 0.60%)	1.13% (1.10% to 1.15%)	<0.05
General symptoms (780–789)		1.60% (1.02% to 2.36%)	1.78% (1.74% to 1.80%)	0.299	Diseases of the veins and lymphatics, and other diseases of the circulatory system (451–459)	0.44% (0.31% to 0.58%)	0.65% (0.63% to 0.67%)	<0.05
Acute respiratory infections (460–466)		1.06% (0.61% to 1.72%)	1.59% (1.55% to 1.61%)	0.052	Neoplasms of unspecified nature (239)	0.42% (0.29% to 0.56%)	0.82% (0.79% to 0.84%)	<0.05
Arthropathies and related disorders (710–719)		–	1.53% (1.50% to 1.56%)	0.01	Persons seeking health services for specific procedures and aftercare (V50–V59)	0.35% (0.24% to 0.48%)	0.57% (0.55% to 0.59%)	<0.05
Other diseases of the digestive system (570–579)		0.79% (0.41% to 1.35%)	1.52% (1.49% to 1.55%)	<0.05	Symptoms (780–789)	–	0.75% (0.73% to 0.77%)	–
Diseases of veins and lymphatics, and other diseases of the circulatory system (451–459)		0.07% (0.00% to 0.37%)	1.52% (1.4% to 1.54%)	<0.05	Disorders of the eye and adnexa (360–379)	–	0.69% (0.66% to 0.70%)	–
Total patients included in these diagnoses		31.40%	30.56%		Total patients included in these diagnoses	38.19%	17.78%	
Total patients (2003)		1503	712 901		Total patients (2003)	9835	630 577	

The results also showed that the type of care provided to the cross-border population was more homogeneous than the care provided to the general population. In the purposive sample, the 10 most frequent diagnoses accounted for 38.19% of the cross-border population, whereas for general population the 10 main diagnoses accounted for only 18.09% of the total. Similarly, in the Catalan case study 26.08% of cross-border patients were covered by the 10 most common diagnoses, whereas only 15.73% of the general population was accounted for by the top 10 diagnoses.

**Points for further research**

To enable further research on this topic it would be necessary:

- To include some mandatory and inter-country equivalent fields in hospitals’ and national healthcare databases as, for example, country of origin, to be able to identify cross-border patients
- To define the data needed to independently identify each of the cross-border categories, and include that data in European databases
- To collect data on a longitudinal basis, to allow analysis of trends regarding volume of cross-border care and possible changes in the type of care

## DISCUSSION

**Summary of findings**

Based on data from hospitals in this study:

- Less than 10% of the hospitals have an agreement for cross-border care
- The main conditions that accounted for cross-border hospitalisation were: diseases of the circulatory system (ischaemic heart disease), injury (fractures) and poisoning, and pregnancy, childbirth and puerperium (normal delivery)
- Cross-border patients were admitted to hospitals with more acute pathologies than the general population (more chronic)
- EU cross-border admissions accounted for less than 1% of all hospitalisations (around 0.07%)
- The volume of cross-border patients seen in emergency departments was almost double that for hospital admissions

In this study we used different methods and types of data which when aggregated provide an estimate of the volume and type of cross-border care provided within Europe, based on information reported by more than 200 hospitals. The main limitation of this study is that the data were retrieved from hospital healthcare information systems, so the validity of the data is dependent on the limitations of these systems.

Some of the limitations we identified were the inability to independently identify foreign patients in the hospitals’ databases, low validity of information in some cases, and the use of different coding systems. Previous studies of cross-border care have also experienced some of these problems.^13^ Other limitations were related to the methods used (such as the fact that one of the two preliminary studies was based on a purposive sample), the limitations inherent in the use of self-reported questionnaires, and possible bias due to the moderately small sample size. However, even in the light of these limitations, the data fulfilled the objectives of the study, since our aim was to provide an estimate of the most common types of patients moving across borders, and of the volume of hospital care these patients represent. Given that this is one of the few studies conducted on volume and type of cross-border care at the European level, the information obtained provides a relevant estimate of this phenomenon, which can inform further studies.

The Catalan case study identified 0.21% of all patients hospitalised as European foreigners. The MARQuIS questionnaire study identified 0.64% of the total admissions in 182 hospitals as European foreign patients. These results are consistent with earlier findings that although the movement of patients across borders has been a reality in Europe for more than 30 years, in terms of numbers it has never been great.^14^ ^15^

However, it is likely that the numbers in these studies were underestimated. Because our data cover only 1 year, we were unable to investigate changes in these numbers with time. A previous study in Belgium has provided data about the changes in this phenomenon: the number of foreign patients treated in Belgium under the E112 system increased from 10 773 in 1998 to 22 477 in 2003,^2^ a change that suggested a major increase in the number of cross-border episodes. The results for volume of cross-border care in the purposive sample studied here were not representative, due to biases inherent in the selection criteria. However, it is interesting to note that the percentage of European patients treated in the emergency services referred to the total population treated was almost double the percentage of elective hospitalisations. As expected, this finding was corroborated by the results of the MARQuIS questionnaire, which adds weight to the construct validity of these studies.

Regarding type of care, diseases of the circulatory system have been identified as the most common reason why European patients are hospitalised abroad. Ischaemic heart disease (mainly acute myocardial infarction) was the most frequent diagnosis in this population. Fractures also ranked near the top of the list of diagnoses for cross-border hospitalisations, and fractures of the lower limbs and skull were the most frequent types. These two ICD groups — diseases of the circulatory systems, and injury and poisoning — were the most important ones for this population. Other common diagnoses identified were deliveries and other diagnoses related to pregnancy, which accounted for completely different group of patients with very specific characteristics and needs. Other acute pathologies such as appendicitis or pneumonia ranked high, as did other diseases of the digestive system, aftercare procedures and disorders of the eye and adnexa.

Other studies that have focused on treatment provided in a specific region yielded findings not unlike ours. Hospitals in the Grand Duchy of Luxembourg recorded the main causes of hospitalisation of foreign patients in 2002 as injuries (16%); disorders of the osteoarticular system, muscles and conjunctional tissue (12.9%); and cases related to pregnancy and birth (11.4%).^16^ In a Belgian case study with data from the year 2002, the main ICD diagnoses for European foreign admissions were chemotherapy, procreative management, coronary atherosclerosis and follow-up examination after surgery.^3^ The type of care identified in the Belgian study seems to differ clearly from the results of the present study, and many factors might have influenced these differences. Comparing Belgium with Catalonia (both studies are based on the total population), Belgium had more than 14 cross-border agreements for the treatment of patients in 2003, whereas Catalonia only had one,^5^ and Catalonia received 20.4 million foreign visitors^17^ (6.8 million population) that year, whereas Belgium had 6.6 million^18^ foreign visitors (10.4 million population). Although more variables could be included in the analysis, it appears that two different categories of cross-border care were investigated in these studies. Further research on this topic should independently address the characteristics of each category of cross-border care, since the diagnoses in each category seem to be different, and therefore the circumstances and needs of patients in different groups would be expected to differ.

Our analysis of the differences in cross-border versus general care confirms the hypothesis that the type of care provided to hospitalised cross-border patients seems to be more homogeneous than the care provided to the general population. The data also showed that cross-border patients were hospitalised for more acute pathologies than the general population, which was characterised by a greater mix of chronic and acute diagnoses upon hospital admission. These differences have economic implications, as in most cases payment is based on admission and not on the type of care provided. Because cross-border patients are hospitalised mainly for more acute pathologies than the general population, and the former are likely to consume more staff time for information and support, the real cost of treatment for cross-border patients may well be higher than the cost of treatment for the general population. Possible differences in costs according to different diagnoses should be taken into account by European policy makers.

The information provided by this study could be used by policy makers in efforts to regulate cross-border care. Our findings concerning type of care may also be helpful for hospitals in their efforts to adapt the care provided to the needs of foreign patients, specifically for the management of the pathologies we have identified as being encountered most frequently.
